# Inferior Parietal Lobe Activity Reveals Bimanual Coupling and Interference

**DOI:** 10.1002/hbm.70172

**Published:** 2025-02-27

**Authors:** Costanza Iester, Monica Biggio, Sabrina Brigadoi, Ambra Bisio, Giampaolo Brichetto, Simone Cutini, Laura Bonzano, Marco Bove

**Affiliations:** ^1^ Department of Neuroscience, Rehabilitation, Ophthalmology, Genetics, Maternal and Child Health University of Genoa Genoa Italy; ^2^ Department of Developmental and Social Psychology University of Padova Padua Italy; ^3^ Department of Experimental Medicine, Section of Human Physiology University of Genoa Genoa Italy; ^4^ Italian Multiple Sclerosis Foundation Scientific Research Area Genoa Italy; ^5^ IRCCS Ospedale Policlinico San Martino Genoa Italy

**Keywords:** bimanual coupling, functional near‐infrared spectroscopy, motor dual‐task, parietal cortex

## Abstract

When humans move both hands simultaneously, bimanual coupling or interference can occur. The circles‐lines paradigm is used to study the bimanual coupling and interference effects: Participants simultaneously draw either lines or circles with both hands (congruent), or draw lines with one hand and circles with the other hand (incongruent condition). Despite extensive behavioral research on bimanual coupling with this paradigm, our knowledge of the neural circuitry involved remains limited. Here, we capitalized on the advantages provided by functional near‐infrared spectroscopy to unveil the neural substrates of bimanual coupling within an ecologically valid experimental setting. Behavioral results confirmed previous literature, showing that the shapes become more oval due to the interference between the hands, causing the circle to resemble a line and vice versa. Additionally, performance in the congruent condition correlated with performance in the incongruent condition. From a neural perspective, we observed greater activity in sensorimotor areas and the right premotor area during the incongruent compared to the congruent condition. A novel temporal analysis of the time course of oxyhemoglobin signals revealed that the right hemisphere reached maximum amplitude before the left during the incongruent condition and revealed differences between conditions in parietal areas, showing that bimanual interference is associated not only with motor areas but also with associative areas. Finally, right inferior parietal lobe activity correlated with bimanual performance, suggesting a role for this area in bimanual tasks when the motor program of one hand is influenced by sensorimotor information from the contralateral hand.

## Graphical abstract

Our study demonstrates fNIRS effectiveness in investigating bimanual interference during the circles‐lines paradigm. fNIRS allows correct drawing positions, ensuring ecological validity. Performance in congruent tasks predicts incongruent performance. Time‐course analysis shows parietal differences, with the right hemisphere peaking earlier during the incongruent condition. Right inferior parietal lobe activity correlates with bimanual interference.

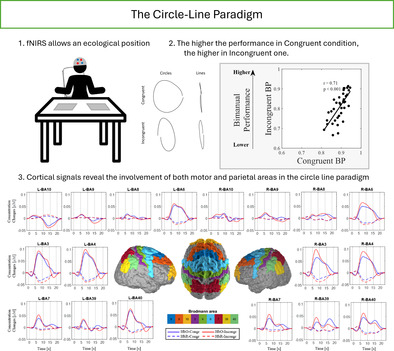

**Summary**

- Functional near‐infrared spectroscopy (fNIRS) enables the investigation of cortical correlates of bimanual performance during the circles‐lines paradigm, offering an ecologically valid approach by allowing correct participants' drawing posture compared to other neuroimaging techniques.
- fNIRS reveals time‐course differences in oxyhemoglobin concentration between congruent and incongruent conditions in parietal areas, indicating that bimanual performance is associated with activity in associative areas, with the right hemisphere peaking earlier during incongruent tasks.
- Right inferior parietal lobe activity correlates with bimanual performance, suggesting its role in effectively evaluating sensorimotor information from the contralateral hand and integrating it into the motor plan in a way that can benefit performance.

## 1 Introduction

In everyday life, executing more than one task at the same time, instead of carrying them out separately, results in decreased performance, which is even worse when the tasks revolve around the same domain. For instance, we might have little trouble walking while having a conversation, but listening to a podcast would be very detrimental to our conversation because the same resources would be shared between activities. This phenomenon, named dual‐task interference, is a very popular topic in cognitive science and can be observed even between simple tasks (for a review see Pashler et al. 1994).

Dual‐task interference also occurs when both tasks involve the motor domain. For example, performing two different motor tasks simultaneously with both hands results in reciprocal interference. The task performed with each hand is negatively affected by the one performed with the other hand, resulting in a mutual deterioration in the performance of each individual hand. A behavioral paradigm that explores dual‐task interference was introduced by Franz et al. (1991) and consisted of drawing two different shapes with both hands: continuous circles with one hand and continuous lines with the other hand. The most interesting outcome of this paradigm is that both the circles and lines assume a more ovalized shape due to the interference from the other hand drawn. Investigating the neural mechanisms underlying motor dual‐tasks, as in bimanual coordination, provides insight into how the brain manages the simultaneous use of both hands, whether they cooperate or interfere with each other. Kennedy et al. (2016) explored bimanual force control, showing that one limb's performance can significantly influence the other. Rudisch et al. (2023) further contributed to this field by examining the neural correlates of bimanual coordination by means of electroencephalography (EEG), highlighting the role of left frontocentral regions in task‐dependent activation and connectivity. Similarly, Rueda‐Delgado et al. (2017) found that increased task difficulty leads to greater long‐range beta band synchronization, suggesting that neural communication across brain regions increases as the task becomes more challenging. Serrien et al. (2004) documented distinct cortico‐cortical coupling patterns during dual‐task performance, indicating that specific neural networks are recruited to manage the complexities of simultaneous motor tasks. By using fMRI, different studies observed larger brain activity for incongruent (when hands perform two different motor tasks simultaneously) than for congruent actions (De Jong et al. 2002; Sadato et al. 1997; Ehrsson et al. 2002; Wenderoth et al. 2004, 2005a, 2005b). In particular, Garbarini et al. (2014) showed that during the execution of the Circles‐Lines bimanual task (Franz et al. 1991) a prefrontal–parietal network, mostly involving the supplementary motor area and the posterior parietal cortex (PPC), was significantly more active in incongruent (circles with one hand and lines with the other hand) than in congruent conditions.

Together, these studies reveal both behavioral outcomes and the neural mechanisms supporting effective dual‐task performance. Moreover, they show that both EEG and fMRI can be used with reasonable proficiency in this research field, but at the same time, both techniques are hindered by crucial limitations. fMRI, while offering high spatial resolution, restricts participants' movement, limiting natural hand movements and being influenced by body posture within the scanner (Bisio et al. 2016). Conversely, EEG allows more freedom of movement but is highly prone to motion artifacts that can distort the data. Importantly, EEG also has lower spatial resolution compared to fMRI, which allows for more precise localization of brain activity. Although fMRI has superior spatial resolution, it poses ecological limitations.

Crucially for the purpose of the present study, these issues can be tackled by using functional near‐infrared spectroscopy (fNIRS), a neuroimaging technique that imposes negligible constraints on participants (both in physical and motor terms), allowing participants to freely move while providing an ecologically sound experimental setting. Although fNIRS does not provide the same level of spatial resolution offered by fMRI and the same level of temporal resolution offered by EEG, it is able to gather information on cortical activity with a much higher temporal resolution than fMRI and a much higher spatial resolution than EEG, thus providing a unique trade‐off between spatial and temporal resolution (see Cutini et al. 2012, for a review). Moreover, EEG and fNIRS provide distinct types of data, with fNIRS offering complementary insights to EEG by capturing different physiological processes. While the Circles‐Lines bimanual task has been extensively studied at the behavioral level, the knowledge about the neural circuitry involved in this task is less robust. Here, we sought to explore the neural substrates of this bimanual task using fNIRS. In the present study, we revisited the “Circles‐Lines” paradigm (Franz et al. 1991), employing two congruent (both hands draw the same shape, either circles or lines) and two incongruent (hands draw different shapes) conditions. We recorded hemodynamic activity with fNIRS from sensorimotor, parietal, and frontal brain regions (which have been shown to be implicated in bimanual tasks [Lucci et al. 2014], see Table 1) while right‐handed participants performed the task of drawing the required shapes while being comfortably seated on a chair and blindfolded. Thus, the aim of the study was to gain a deeper understanding of the mechanisms underlying bimanual coordination during congruent and incongruent tasks in an ecological setting using fNIRS. Congruent conditions generally facilitate more accurate responses due to bimanual coupling, while incongruent conditions often generate bimanual interference that decreases movement accuracy. By exploring the brain activity during this task, we might provide insights about how the brain manages competing demands and which specific brain areas are engaged in resolving potential conflicts between bimanual actions. Thanks to the higher temporal resolution of fNIRS, we could perform a more fine‐grained temporal analysis compared to what could have been performed with fMRI, which enabled us to illustrate in detail how cortical responses evolve over time during the bimanual task. Finally, a time‐to‐peak analysis was conducted to provide new insights into the neural dynamics of bimanual coordination, with the expectation that incongruent tasks would require a different temporal pattern to manage separate motor programs for each hand.

**TABLE 1:** Description of the recording channels: Optode placements based on the 10–10 system, MNI coordinates, cortical regions, and Brodmann's areas.

Channel	Label_S	Label_D	*x* coordinate	*y* coordinate	*z* coordinate	Hemisphere	Lobe	Anatomical location	BA
1	CP1	C1	−27	−36	71	Left	Parietal	Postcentral gyrus	3
2	C3	C1	−42	−20	62	Left	Frontal	Precentral gyrus	4
3	FC1	FC3	−38	12	55	Left	Frontal	Middle frontal gyrus	6
4	FC1	FCz	−13	12	67	Left	Frontal	Superior frontal gyrus	6
5	FC1	C1	−26	−5	68	Left	Frontal	Superior frontal gyrus	6
6	C3	FC3	−50	−3	50	Left	Frontal	Precentral gyrus	6
7	Cz	C1	−17	−20	74	Left	Frontal	Precentral gyrus	6
8	CP1	P1	−24	−62	62	Left	Parietal	Superior parietal lobule	7
9	CP1	CPz	−16	−50	72	Left	Parietal	Postcentral gyrus	7
10	P3	P1	−32	−73	47	Left	Parietal	Superior parietal lobule	7
11	Pz	P1	−13	−73	56	Left	Parietal	Superior parietal lobule	7
12	FC1	F1	−23	26	56	Left	Frontal	Superior frontal gyrus	8
13	F3	F1	−31	39	41	Left	Frontal	Middle frontal gyrus	9
14	F3	FC3	−45	25	41	Left	Frontal	Middle frontal gyrus	9
15	Fz	F1	−9	41	50	Left	Frontal	Superior frontal gyrus	9
16	Fpz	Fp1	−12	67	0	Left	Frontal	Medial frontal gyrus	10
17	AF3	Fp1	−24	63	9	Left	Frontal	Middle frontal gyrus	10
18	AF3	AFz	−12	62	23	Left	Frontal	Superior frontal gyrus	10
19	AF3	F1	−23	52	32	Left	Frontal	Superior frontal gyrus	10
20	P3	CP3	−46	−61	46	Left	Parietal	Inferior parietal lobule	39
21	C3	CP3	−52	−34	52	Left	Parietal	Postcentral gyrus	40
22	CP1	CP3	−39	−48	60	Left	Parietal	Inferior parietal lobule	40
23	CP2	C2	27	−35	71	Right	Parietal	Postcentral gyrus	3
24	C4	C2	42	−21	62	Right	Frontal	Precentral gyrus	4
25	Cz	C2	17	−21	75	Right	Frontal	Precentral gyrus	6
26	FC2	FCz	14	13	66	Right	Frontal	Superior frontal gyrus	6
27	FC2	FC4	39	12	54	Right	Frontal	Middle frontal gyrus	6
28	FC2	C2	27	−4	68	Right	Frontal	Superior frontal gyrus	6
29	C4	FC4	52	−4	48	Right	Frontal	Precentral gyrus	6
30	Pz	P2	15	−73	57	Right	Parietal	Superior parietal lobule	7
31	P4	P2	33	−74	48	Right	Parietal	Superior parietal lobule	7
32	CP2	CPz	17	−50	73	Right	Parietal	Postcentral gyrus	7
33	CP2	P2	25	−62	63	Right	Parietal	Superior parietal lobule	7
34	FC2	F2	24	26	55	Right	Frontal	Superior frontal gyrus	8
35	Fz	F2	10	41	50	Right	Frontal	Superior frontal gyrus	9
36	F4	F2	30	40	41	Right	Frontal	Middle frontal gyrus	9
37	F4	FC4	44	25	40	Right	Frontal	Middle frontal gyrus	9
38	Fpz	Fp2	13	67	0	Right	Frontal	Medial frontal gyrus	10
39	AF4	AFz	13	61	24	Right	Frontal	Superior frontal gyrus	10
40	AF4	Fp2	25	63	9	Right	Frontal	Middle frontal gyrus	10
41	AF4	F2	22	52	33	Right	Frontal	Superior frontal gyrus	10
42	P4	CP4	46	−62	47	Right	Parietal	Inferior parietal lobule	39
43	CP2	CP4	39	−49	60	Right	Parietal	Postcentral gyrus	40
44	C4	CP4	52	−35	52	Right	Parietal	Postcentral gyrus	40

## 2 Methods

### 2.1 Participants

Thirty‐eight healthy volunteers (23 females; mean age ± SD: 32.4 ± 12.7 years) were enrolled in the study. All participants were right‐handed, as determined by the Edinburgh Handedness Inventory (Oldfield 1971). None of the participants had a history of orthopedic or neurological illness, nor did they exhibit any motor or sensory deficits related to the upper limb. The experimental protocol received approval from the ethics committee of Azienda Ospedaliera “San Martino,” Genoa, Italy (P.R. 271REG2017) and was conducted following legal requirements and international standards (Declaration of Helsinki, 1964). All subjects provided written informed consent to participate in the study.

### 2.2 Experimental Procedure

Participants underwent the Circles‐Lines Paradigm while being monitored with fNIRS. They were seated on a chair behind a table and asked to draw continuous circles or vertical lines with both hands while blindfolded. The left hand drew on an Apple iPad Pro (12.9″) placed on the left of the participant's sagittal midline, while the right hand drew on a drawing board on the right of the participant's sagittal midline. The drawing board provided the same drawing sensation for both hands, but only the data from the left hand on the Apple iPad Pro were recorded. Participants had to draw continuously, concurrently with the two hands, in four experimental conditions: drawing lines with both hands (LL), drawing circles with both hands (CC), drawing lines with the left hand and circles with the right (LC), and drawing circles with the left hand and lines with the right (CL). When participants drew the same figures with both hands, they performed a congruent condition (i.e., LL and CC conditions). Instead, when they drew different shapes, they performed an incongruent condition (i.e., LC and CL conditions). The Lines condition included tasks in which the left hand drew only lines (i.e., LL and LC conditions), while the Circles condition included tasks in which the left hand drew only circles (i.e., CC and CL conditions). Conditions were presented in a randomized order, and 10 trials were collected for each condition, resulting in 40 trials in total. The choice of 10 trials per condition was based on established practices in the literature. This number was deemed sufficient to ensure reliable behavioral (e.g., Franz et al. 1991; Garbarini et al. 2014) and fNIRS (Curzel et al. 2021; Lloyd‐Fox et al. 2010; Zhao et al. 2020; Zhou et al. 2022) measures. Additionally, the seated task setup and absence of vocal responses minimized potential artifacts, further supporting the reliability of the chosen trial count. Increasing the number of trials could risk introducing fatigue or adaptation effects, making 10 trials a balanced and robust choice for this study design. Both the task duration and the rest duration were jittered to avoid expectation (task duration = 10/12 s; rest duration = 15 ± 2 s). Participants were instructed to draw in time with a metronome (*f* = 0.6 Hz): each sound of the metronome corresponded to either a complete circle or a line drawn back and forth. During the entire experiment, participants wore the fNIRS cap. The montage was performed at the beginning, and the average setup time took around 30 min.

### 2.3 fNIRS Assessment

The fNIRS signals were acquired using a portable, multichannel NIRS system (NIRSport 2, NIRx Medical Technologies, Berlin, Germany). The instrument allowed the calculation of changes in the concentration of HbO and HbR in measurement channels. Pairs of sources and detectors operating at two continuous wavelengths of near‐infrared light (760 and 850 nm) generated channels. All optodes were placed on a soft black tissue cap (EasyCap, Germany) worn by the participant. To ensure correct channel placement, an fNIRS cap with EEG references was used. The participant's head circumference was measured to select the cap size, and the Vertex (Cz in EEG references) was located at the midpoint between the Nasion, Inion, and the ear tragus points for accurate positioning. The system consisted of 16 LED illumination sources and 16 active detectors arranged to form 44 standard channels (3 cm) and 8 short‐separation (SS) channels (8 mm). These channels covered the frontal, premotor, motor, sensory and parietal brain areas. A detailed description of the standard channels, their MNI coordinates, and the corresponding Brodmann's area (BA) can be found in Table 1. The anatomical localizations of the channels were determined using the Brodmann brain atlas in the fNIRS Optodes' Location Decider (fOLD) toolbox (Zimeo Morais et al. 2018). The sampling frequency was set at 8.7 Hz.

### 2.4 Behavioral Data Analysis

**FIGURE 1:**
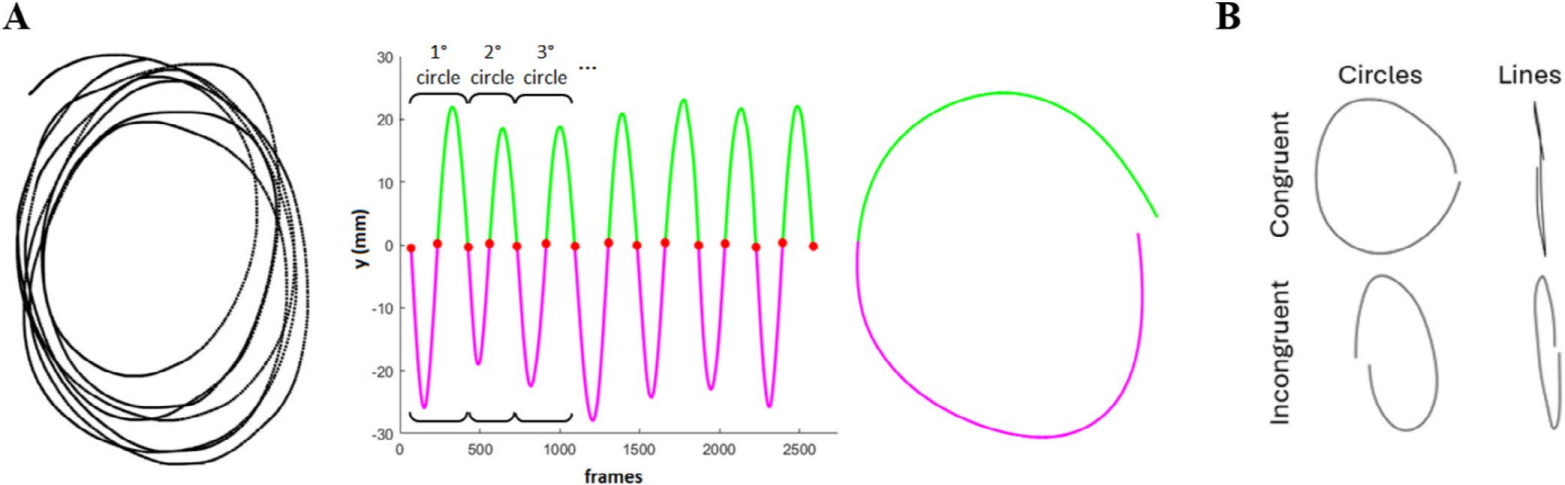
(A) Results of the steps of the zero‐crossing algorithm for the segmentation of the figures. On the left, the *x*‐*y* trajectories drawn by a sample participant in a trial in the circles–circles condition (left hand). The middle panel shows the corresponding *y* trajectories (*y* axis) over the frames (*x* axis). The red dots indicate the zero‐crossing points automatically identified by the algorithm. The magenta lines indicate the *y* trajectory below these points and the green lines indicate the *y* trajectory above these points. A pair of magenta and green lines represents the *y* trajectory for a single circle. On the right is an example of the result of the zero‐crossing algorithm segmentation. (B) Examples of a participant's trajectories in the four conditions.

Data from the left hand were collected through the Apple iPad Pro. A custom‐made MATLAB software (Biggio et al. 2021) was used to analyze the left‐hand data. Through a semi‐automated procedure, in each trial the written trace was segmented into circles or reciprocal lines, and then the ovalization index (OI) was computed. In particular, the onset of each trial was manually identified by the experimenter in order to exclude spurious data accidentally captured by the iPad that was not part of the written trace. The trace was then automatically segmented using a zero‐crossing algorithm applied to the *y* coordinate. This allowed the identification of the different figures (both circles and reciprocal lines) within each trial (Biggio et al. 2021). The different steps of the analysis are shown in Figure 1. Then, for each figure, four points were automatically identified: the first two zero crossings, the negative peak, and the positive peak. The distances between the points at the zero crossings and the distances between the points at the peaks were then performed. The ratio of these two distances was computed using the higher number as the denominator. OI was performed as the average of these ratios among figures belonging to the same condition. Thus, the OI was within the range [0, 1], with 0 indicating perfect reciprocal lines and 1 a perfect circle. The OI allowed quantifying the distortion of the trajectory from the perfect figure. To compare the congruent and incongruent conditions for both the Circles and Lines conditions, for the Lines condition we subtracted the OI values from 1 (i.e., 1‐OI), so that for both Lines and Circles condition 1 would indicate the perfect shape. After averaging OIs in the congruent (CC, LL) and the incongruent (CL, LC) conditions, we obtained two behavioral parameters for each participant. These parameters will refer to bimanual performance (BP). A higher value indicates better performance, which translates to higher bimanual coupling in the congruent condition and lower interference in the incongruent condition. According to the Anderson–Darling test, all the experimental data followed a normal distribution. A repeated measures ANOVA with CONGR (2 levels: Congruent, Incongruent) and FORM (2 levels: Line, Circle) as within‐subject factors was performed. Furthermore, the Pearson correlation coefficient was computed between the two conditions. Then, two groups were extracted using a data‐driven approach (i.e., cluster analysis: k‐means, two groups) on the correlation coefficients. To ensure the groups were homogeneous, we conducted an unpaired *t* test to check for age differences and applied Fisher's exact test to assess gender proportions between the groups. We chose k‐means clustering for the group division because it is a robust, data‐driven approach that clusters data by minimizing within‐cluster variance, ensuring individuals with similar performance patterns are grouped together. To perform the analysis, we used MATLAB's k‐means function and selected the squared Euclidean distance as the distance metric. For the selection of cluster centers, the kmeans algorithm by default initializes the centroids using the “k‐means++” method, which improves the convergence rate by choosing initial cluster centers that are well spread out. Regarding the number of iterations, the algorithm ran until convergence or for a maximum of 100 iterations.

Moreover, BP mean values were subjected to one‐way mixed ANOVA with Condition (two levels: Congruent, Incongruent) as within‐subjects factors and Group (two levels: HBP, LBP) as between‐subjects factors. Significant interactions in the ANOVA were followed by post hoc Bonferroni‐corrected tests.

Statistical analyses were performed with STATISTICA (StatSoft Inc., 2011, STATISTICA, Data Analysis Software System).

### 2.5 fNIRS Data Analysis

For each participant, the fNIRS signal was preprocessed using MATLAB (MathWorks, MA, USA) with in‐house scripts and some of the Homer3 NIRS processing package functions (Huppert et al. 2009). Channels with low signal‐to‐noise ratios or with a very low optical intensity were discarded (SNR < 2); the intensity data of the survived channels were converted to optical density changes. Motion artifacts were corrected by applying the wavelet motion correction technique (iqr = 0.5) (Molavi and Dumont 2012). Then, residual motion artifacts were identified by the *hmrR_MotionArtifact* function. If the identified motion artifacts were within −5 and 10 s post task onset, the trial in question was discarded from further analysis. A band‐pass filter (0.01–3 Hz) was applied to remove slow drifts and very high frequencies. An age‐dependent differential pathlength factor was computed for each participant (Scholkmann and Wolf 2013), and then both the HbO and the HbR concentration changes were computed through the modified Beer–Lambert law (Delpy 1988). The mean HRF for each task block, participant, and channel was recovered using a General Linear Model (GLM) approach. The GLM was solved using an iterative weighted least squares approach (Barker et al. 2013). Temporal basis components for the hemodynamic response function (HRF) consisted of a consecutive sequence of Gaussian profiles with a spacing and standard deviation of 2 s. The block average interval for the hemodynamic response computation was set from −2 to 23 s. To remove the physiological noise, an additional regressor in the GLM was added. For each standard channel, the most correlated SS channel was used as aregressor.

Then, the HRF of channels belonging to the same BA was averaged, thus obtaining the HRFs of 18 regions of interest (Table 1).

### 2.6 fNIRS Global Analysis

For each participant, condition, and BA, the average of the HbO/HbR mean hemodynamic responses in the range between 5 and 17 s after stimulus onset was computed and chosen as a metric for statistical analyses. Active BAs were defined as those regions with statistically significant positive changes in HbO concentration (one‐tail *t* test, FDR correction for multiple comparisons) and statistically significant negative changes in HbR concentration during the task, compared to zero (one‐tail *t* test, FDR correction for multiple comparisons). Further statistical analyses were performed only on the BA found to be active in at least one condition. To compare the four different conditions, concentration changes were analyzed by means of repeated measures ANOVA with CONGR (2 levels: Congruent Incongruent), FORM (2 levels: Line, Circle) and BA (13 levels: active BAs) as within‐subject factors. Post hoc analysis was performed using Fisher's least significant difference (LSD) test. Statistical analyses were performed with STATISTICA (StatSoft Inc., 2011, STATISTICA, Data Analysis Software System).

### 2.7 fNIRS Temporal Analysis

For the first time, two temporal analyses exploiting the characteristics of the fNIRS signal were developed and applied, allowing us to investigate brain dynamics. First, to understand the evolution of hemodynamic activity over the course of execution and provide valuable insights on the unfolding of the process and the neural system involved, a temporal bin analysis was performed. We divided the entire temporal period of the HRF into four 5 s‐windows (0–5; 5–10; 10–15; 15–20 s; where 0 s corresponds to the beginning of the task) to unveil whether activity patterns differed in different temporal ranges. For each range, active BAs were calculated following the procedure detailed above, and then the average HRF within the temporal window was submitted to repeated measures ANOVA with CONGR (2 levels: Congruent Incongruent), FORM (2 levels: Line, Circle), BA (13 levels: active BAs), and BIN (3 levels: 5–10; 10–15; 15–20 s) as the first within‐subject factor. Range 0–5 s revealed no active BAs; thus, it was neglected from further analysis. Then, we focused our attention on the 5–10 s window, the one which reveals the early hemodynamic response related to cortical activity. This analysis allowed us to reveal potential additional areas that exhibited differences in activity between the congruent and incongruent tasks.

Second, we performed an even finer temporal analysis to find the time‐to‐peak activation for each BA. For each sample, condition, and BA, we compared the hemodynamic responses of individual subjects, both HbO and HbR, after stimulus onset to zero using two one‐sample *t* tests (one for positive and one for negative responses, HbO and HbR, respectively). This allowed us to identify active BAs in each condition and frame (we used the FDR multiple comparison correction). For each BA, the active frame range started with the first frame (after the stimulus onset) and concluded with the last frame (by the 20th s), for which the BA was found active in at least one condition for both HbO and HbR concentration changes. These active frame ranges were used for the time‐to‐peak analysis. The time‐to‐peak of a function is the time at which the function reaches its maximum value. For each participant, condition, and BA, the maximum positive value of the HbO HRF was detected in its active frame range and the corresponding time was saved. Then, to compare the time‐to‐peak values, a repeated measures ANOVA with CONGR (2 levels: Congruent, Incongruent), FORM (2 levels: Line, Circle), and BA (13 levels: active BAs) as a within‐subject factor was performed. In this case, the active BAs are the ones that have at least one active frame in one condition. Statistical analyses were performed with STATISTICA (StatSoft Inc., 2011, STATISTICA, Data Analysis Software System).

### 2.8 fNIRS And Behavioral Analysis

To explore a possible relationship between hemodynamic activity and behavioral data, we correlated the BP index with the BAs that were found to be statistically active in the global analysis. For each of these BAs, we calculated the Pearson correlation coefficient, and we applied the Bonferroni correction method for multiple comparisons. Then, BAs with a significant correlation with the BP index were compared between the two groups using an unpaired *t* test.

## 3 Results

### 3.1 Behavioral Analysis

**FIGURE 2:**
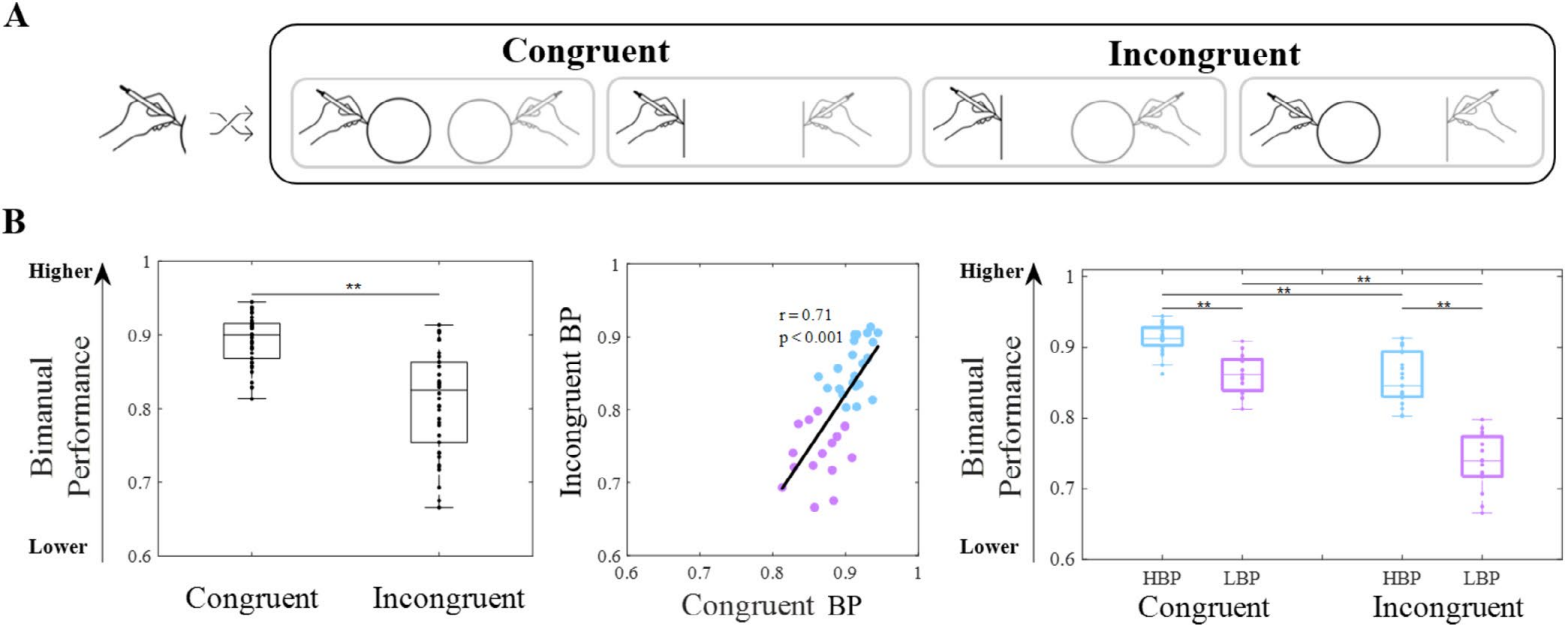
(A) Experimental paradigm: The congruent conditions, while both hands draw the same shape and the incongruent conditions, while the two hands draw different shapes. (B) Behavioral results: On the left side, statistical difference between congruent and incongruent conditions; in the middle, the correlation between the conditions, light‐blue dots represent the high bimanual performance—HBP group, while the purple dots represent the low bimanual performance—LBP group; on the right side, statistical differences within and between subgroups.

The results indicated that performance was higher during the congruent task compared to the incongruent one (Congruent vs. Incongruent, 0.89 vs. 0.81, *t*_(37)_ = 9.97, *p* < 0.001). Furthermore, by correlating the behavioral data obtained during the congruent and incongruent tasks, it was observed that participants who revealed a high performance in the congruent task also exhibited a high performance in the incongruent task (*r* = 0.71, *p* < 0.001). Then, the k‐means algorithm highlighted: one group with higher BP in both conditions (high bimanual performance—HBP) (23 participants, 16 females; mean age ± SD: 31.7 ± 9.8 years) and another group with lower BP in both conditions (low bimanual performance—LBP) (15 participants, 7 females; mean age ± SD: 35.9 ± 16.3 years) (Figure 2B). No significant differences were found in age (*p* = 0.39) or in gender proportions between the groups (*p* = 0.19). Investigating differences among groups and conditions (mixed ANOVA; between groups, two levels: HBP, LBP; within groups, two levels: Congruent, Incongruent), we found a statistical difference between groups (HBP>LBP, *F*_(1,36)_ = 105.57, 0.88 vs. 0.80, *p* < 0.001) and conditions (Congruent >Incongruent, *F*_(1,36)_ = 200.98, 0.89 vs. 0.81, *p* < 0.001). Moreover, a significant interaction was found (*F*_(1,36)_ = 29.95, *p* < 0.001). For each group, Congruent BP was significantly higher than Incongruent BP (HBP, 0.91 vs. 0.86; LBP, 0.86 vs. 0.74; *p* < 0.001) and, for each condition, HBP was significantly higher than the LBP group (Congruent, 0.91 vs. 0.86, *p* < 0.001; Incongruent, 0.86 vs. 0.74, *p* < 0.001) (see Figure 2B). We also performed a *t* test on the delta values in order to highlight in detail the core of the interaction between the group and the condition: for each group, we subtracted the BP value in the incongruent condition from the BP value in the congruent condition. This *t* test revealed that the performance of the LBP group was more negatively affected by congruency, with a delta BP equal to 0.11, than the HBP group, with a delta BP equal to 0.06 (*t*_(36)_ = 2.31, *p* = 0.03).

### 3.2 fNIRS—Global Analysis

**FIGURE 3:**
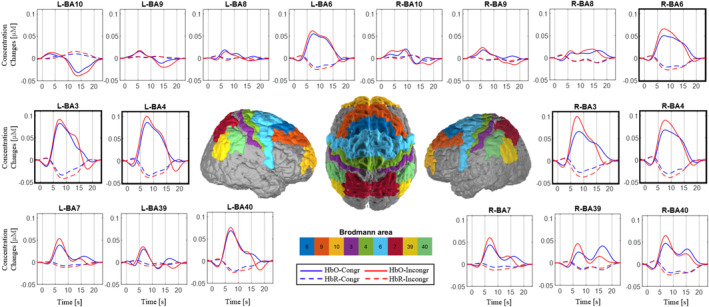
The boxes show variations in HbO (continuous lines) and HbR (dashed lines) concentrations during the congruent (blue lines) and incongruent (red lines) conditions, respectively. Left hemisphere (L‐), right hemisphere (R). Black lines define bins. The correspondence of BAs on the brain are shown. Boxes with bold borders represent BAs with a statistically significant difference between congruent and incongruent conditions in the global analysis.

As for behavioral data, hemodynamic activity was analyzed in detail as well. Thirteen BAs out of the 18 were found to be active. Exploring differences in changes in HbO concentration, a significant main effect of the BA factor (*F*_(12,444)_ = 14.74, *p* < 0.001) and the interaction CONGR*BA (F_(12,444)_ = 1.89, *p* = 0.034) were found. The interaction effect revealed that L‐BA4, R‐BA4, L‐BA3, R‐BA3, and R‐BA6 showed significantly stronger activation in the incongruent condition compared to the congruent one (L‐BA4, 0.065 vs. 0.058 μM, *p* = 0.029; L‐BA3, 0.059 vs. 0.052 μM, *p* = 0.031; R‐BA6, 0.048 vs. 0.041 μM, *p* = 0.029; R‐BA4, 0.060 vs. 0.051 μM, *p* = 0.008 and R‐BA3, 0.060 vs. 0.048 μM, *p* < 0.001) (Figure 3) (see Supporting Information for HbR concentration changes results).

### 3.3 fNIRS—Temporal Analysis

In the bin analysis, 14 BAs were found to be active in at least one bin, whereas L‐BA10, L‐BA9, L‐BA8, and R‐BA10 never resulted active and were, therefore, excluded from the bin analysis. Exploring differences between conditions for HbO concentration changes in the 5–10 s bin, a significantly greater value in the incongruent compared to the congruent condition (BIN* CONGR *BA, *F*_(26,962)_ = 2.21, *p* < 0.001) was found again in L‐BA4 (0.087 vs. 0.075 μM, *p* < 0.001), L‐BA3 (0.079 vs. 0.071 μM, *p* = 0.03), R‐BA6 (0.04 vs. 0.03 μM, *p* < 0.001), R‐BA4 (0.06 vs. 0.046 μM, *p* < 0.001), R‐BA3 (0.079 vs. 0.06 μM, *p* < 0.001), but also in L‐BA7 (0.085 vs. 0.056 μM, *p* = 0.01), R‐BA7 (0.047 vs. 0.035 μM, *p* < 0.001), R‐BA39 (0.031 vs. 0.019 μM, *p* < 0.001), and R‐BA40 (0.052 vs. 0.04 μM, *p* < 0.001) (see Supporting Information for HbR concentration changes results).

### 3.4 fNIRS—Time‐to‐Peak Analysis

**FIGURE 4:**
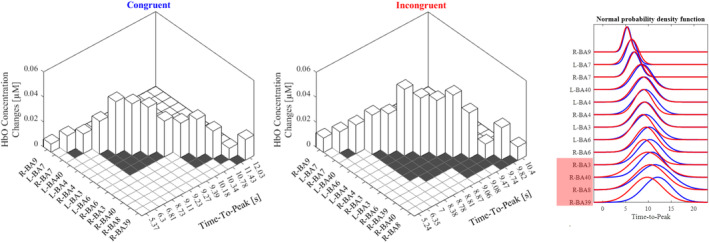
On the left, the figure summarizes the findings from this study. The height represents the average concentration change of each area during the task. Higher values indicate a greater increase of HbO concentration. The order of the areas reflects the order in which they reached their peak. The boxes indicate whether the areas differ significantly (white boxes) or not (black boxes) between the congruent and incongruent conditions in reaching the peak. On the right, normal probability density function for each condition and active BA. The red box highlights areas which significantly anticipated during the incongruent condition compared to the congruent one. Each curve has the mean value equal to the mean of time‐to‐peak values among participants and the standard deviation equal to the standard deviation of time‐to‐peak values among participants.

Thirteen BAs out of 18 were found to be active looking at the time‐to‐peak analysis. We observed a significant main effect for the factor CONGR (*F*_(1,37)_ = 7.32, *p* = 0.01) and BA (*F*_(12,444)_ = 42.31, *p* < 0.001) and an interaction effect CONGR*BA (*F*_(12,444)_ = 2.97, *p* < 0.001). The CONGR effect revealed that in general the incongruent condition anticipated the congruent one (8.54 vs. 9.15 s, *p* = 0.01). In both conditions, the first area to reach the peak was R‐BA9, followed by R‐BA7 and L‐BA7. In the congruent condition, the remaining brain areas exhibited a distinctive pattern: some areas, including L‐BA40, BA4, L‐BA3, and L‐BA6, show an early peak in activity, while others reach their peak later. In the incongruent condition, however, it appears that after the same initial pattern (R‐BA9 followed by R‐BA7 and L‐BA7), all the remaining areas revealed less significant differences in reaching their peak (see the gray boxes in Figure 4). In fact, during the incongruent task, there was an anticipation of the time‐to‐peak in the right hemisphere areas, specifically R‐BA3 (9.06 vs. 10.35 s, *p* < 0.001), R‐BA40 (9.82 vs. 10.78 s, *p* = 0.013), R‐BA8 (10.40 vs. 11.43 s, *p* = 0.007), and R‐BA39 (9.73 vs. 12.03 s, *p* < 0.001) (see Figure 4).

### 3.5 fNIRS and Behavioral Analysis

**FIGURE 5:**
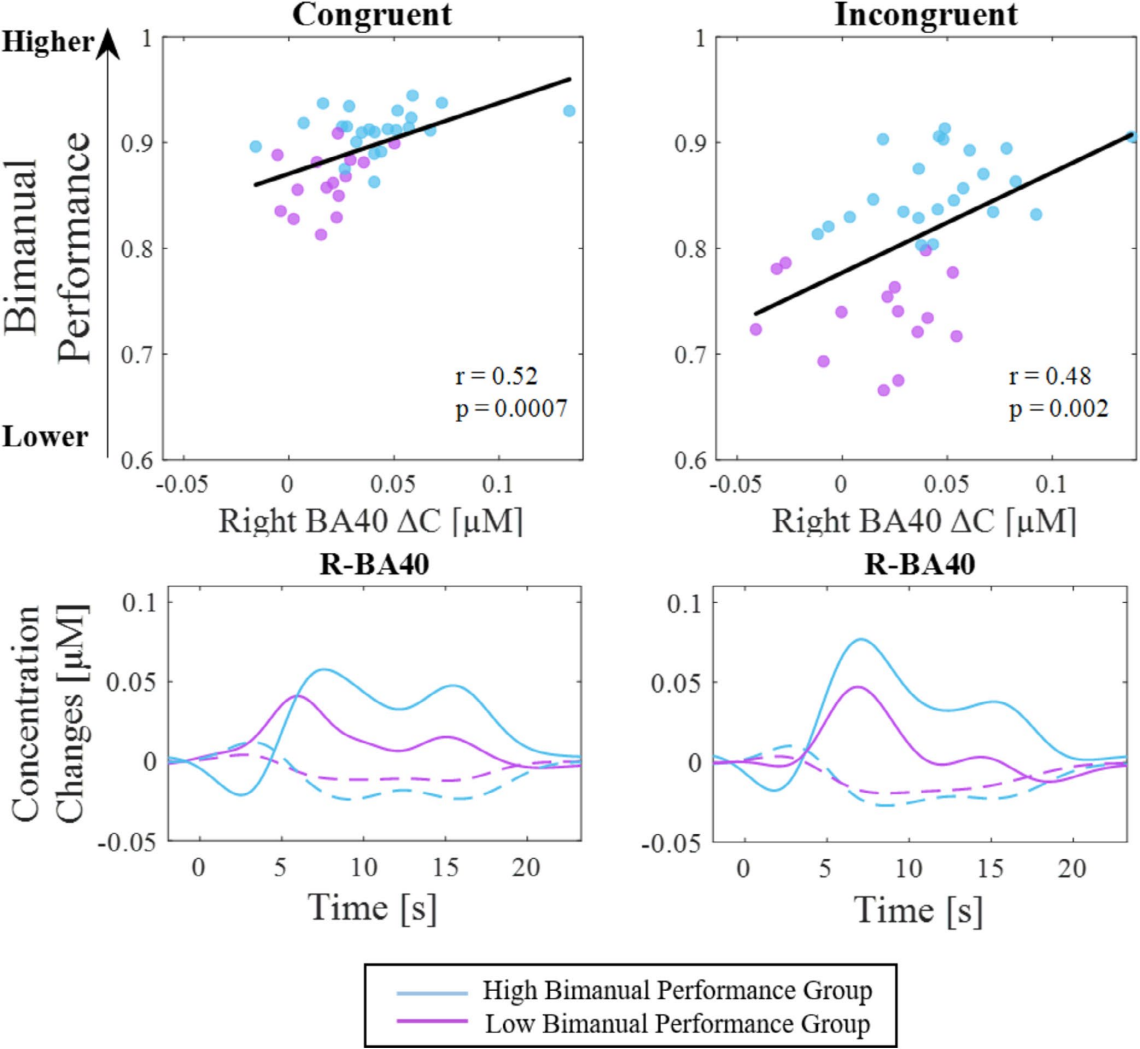
On the top, correlations between R‐BA40 HbO concentration changes and the behavioral index for each condition (on the left side, congruent condition; on the right side, incongruent condition). Purple dots correspond to LBP group, while light blue dots correspond to HBP group. On the bottom, Hemodynamic Response Functions for R‐BA40 for each condition (on the left side, congruent condition; on the right side, incongruent condition). The same colors were used to identify the two groups.

A significant correlation, surviving Bonferroni correction (*p* = 0.004), between the BP and R‐BA40 was found (congruent condition: *r* = 0.52, *p* < 0.003; incongruent condition: *r* = 0.48, *p* < 0.003). Specifically, individuals who activated R‐BA40 more had a higher BP in both the congruent and incongruent tasks (Figure 5). Using the clustering into groups obtained in the behavioral analysis, we compared the fNIRS signal of R‐BA40 between the two groups. The HBP group demonstrated greater activity in R‐BA40 than the LBP group (Figure 5).

## 4 Discussion

As our first result, we found a worse performance (i.e., lower BP) during the incongruent compared to the congruent condition, triggered by a sizeable behavioral interference. This finding demonstrates the influence of one hand on the other during the circles and lines paradigm, which is in line with the literature (Biggio et al. 2021; Franz et al. 1991; Garbarini et al. 2014).

Furthermore, from a behavioral standpoint, it was observed that performance in the congruent condition predicted performance in the incongruent condition. Specifically, participants with HBP in the congruent condition also had higher bimanual performance in the incongruent condition. This suggests that each participant's performance (during the congruent or incongruent condition) reflects their ability to modulate signals from the opposite hemisphere. This observation led usto divide the whole group into two subgroups, one characterized by higher BP and the other by lower BP in both conditions, based on behavioral data, in order to investigate the cortical correlates underlying their performance differences and to determine whether specific cortical resources contribute to the observed differences in bimanual coordination between the two conditions.

From a neural perspective, differences between the congruent and incongruent tasks were also observed. When examining the overall signal, it was noticed that the sensorimotor areas (BA4 and BA3) and the right premotor cortex (R‐BA6) were more active during the incongruent condition. Moreover, the development of the two novel temporal analyses allowed us to unveil new insights. First, the bin temporal analysis highlighted differences between conditions also in parietal areas, particularly in BA7 and in right BA39 and BA40. This allowed us to associate bimanual interference not only with motor areas but also with associative areas. Second, the dynamics of reaching the peak showed that during the incongruent task, the right hemisphere tends to reach the maximum amplitude before the left hemisphere. This result is reported for the first time, and it is conceivable to argue that when both hands are engaged in different tasks, the right hemisphere needs to suppress signals coming from the dominant left hemisphere to assert its own command.

We also speculate that in more complex tasks, the right hemisphere might play a crucial role due to an increased attentional demand. Specifically, Wenderoth et al. (2005b) have shown that higher activity in the precuneus can be explained by continuous shifts of attention across limbs, aiming to monitor their specific trajectories. Finally, considering the complexity of both behavioral and neural data, combining their information together helped us to understand how they are connected. The results indicate greater activity in the right inferior parietal lobe (R‐BA40) when performance was higher, both in the congruent and incongruent conditions. Specifically, the HBP group exhibited higher activity in the right inferior parietal lobe (R‐BA40) compared to the LBP group. This suggests a pivotal role for the right inferior parietal lobe (R‐BA40) in bimanual tasks, confirmed by the fact that the right inferior parietal lobe (R‐BA40) shows a conspicuous activity—sustained during almost the entire duration of the task—which is correlated with behavioral outcomes. We could speculate that this constant demand for oxygen throughout the entire task may be related to a continuous monitoring of behavior: indeed, PPC plays an important role in egocentric coordinate transformation during the whole motor task (Bai et al. 2021; Buneo and Andersen 2006). In humans, the PPC—especially the right one—encodes egocentric information during the perception and exploration of the peripersonal space (Chokron 2003; Sherrill et al. 2015). Structural changes, hypoperfusion, and decreased metabolism of PPC have been shown to undermine the performance of the drawing tests (Matsuoka et al. 2011). Also, decreased regional cerebral glucose metabolism in the right inferior parietal lobe is associated with poor performance on the clock drawing test in patients with Alzheimer's disease (Lee et al. 2008).

From these findings, we could consider the right inferior parietal lobe (R‐BA40) as an area involved in the monitoring of the sensorimotor states, including the integration of intention, action, and sensory feedback (Fink et al. 1999). Indeed, goal‐directed actions necessitate a mechanism that monitors sensorimotor inputs to ensure that motor outputs are congruent with current intentions. The simultaneous execution of two different motor tasks with both hands (i.e., dual motor task) results in a reciprocal interference, inducing a mutual degradation in the performance of each individual hand. It has been shown that the parietal cortex is a crucial hub where different spatial features are integrated, and it might be one of the candidate regions from which interference arises when directionally incompatible movements are performed (Wenderoth et al. 2004). Thus, we can assume that, in this task, the role of the right inferior parietal lobe (R‐BA40) is to detect a possible incongruence in the motor program of one hand influenced by the sensorimotor information coming from the contralateral hand, likely through the callosal communication between homologous areas of the two hemispheres (Biggio et al. 2021). Moreover, it is also worth noting that the inferior parietal lobe (BA40) plays a crucial role in drawing. Consequently, the relationship between BP and BA40 activity may also be linked to the visuomotor nature of the task (see Raimo et al. 2021 for a review).

Interestingly, in a work aimed at studying the functional connectivity during monitoring for visuomotor incongruence, it was found that the right inferior parietal lobe had a strong functional connectivity with the right dorsolateral prefrontal cortex. In this vein, the authors suggested that a cooperative activation of the dorsolateral prefrontal cortex, in coordination with the PPC, is necessary to detect a mismatch when performing an incongruence task (Ohashi et al. 2018).

Consequently, it is conceivable to hypothesize that the activity in the right inferior parietal lobe (R‐BA40), higher in the HBP group with respect to the LBP group, could be transferred to the motor areas (BA4) as a signal to cope with the contralateral hand influence in the dual motor task.

## 5 Conclusion

In conclusion, we demonstrated that fNIRS can overcome the spatial constraints of fMRI that may influence motor performance during the circles‐lines paradigm. For the first time, two different analyses of the time course of the oxyhemoglobin concentration were carried out, exploiting the high sample rate of fNIRS. We found that the right hemisphere reached its oxyhemoglobin concentration peak earlier during incongruent tasks. Furthermore, our results showed that activity in the right inferior parietal lobe (R‐BA40) correlated with BP, suggesting its crucial role in effectively evaluating sensorimotor information from the contralateral hand and integrating it into the motor plan in a way that can benefit performance.

## Conflicts of Interest

The authors declare no conflicts of interest.

## Supporting information


**Data S1.** Supporting Information.

## Data Availability

The original contributions presented in the study are included in the article/Supporting Information, further inquiries can be directed to the corresponding authors.

## References

[hbm70172-bib-0001] Bai, S. , W. Liu , and Y. Guan . 2021. “The Visuospatial and Sensorimotor Functions of Posterior Parietal Cortex in Drawing Tasks: A Review.” Frontiers in Aging Neuroscience 13: 717002. 10.3389/fnagi.2021.717002. 34720989 PMC8551751

[hbm70172-bib-0002] Barker, J. W. , A. Aarabi , and T. J. Huppert . 2013. “Autoregressive Model Based Algorithm for Correcting Motion and Serially Correlated Errors in fNIRS.” Biomedical Optics Express 4: 1366–1379. 24009999 10.1364/BOE.4.001366 PMC3756568

[hbm70172-bib-0003] Biggio, M. , A. Bisio , F. Garbarini , and M. Bove . 2021. “Bimanual Coupling Effect During a Proprioceptive Stimulation.” Scientific Reports 11, no. 1: 15015. 10.1038/s41598-021-94569-8. 34294818 PMC8298576

[hbm70172-bib-0004] Bisio, A. , L. Pedullà , L. Bonzano , P. Ruggeri , G. Brichetto , and M. Bove . 2016. “Evaluation of Handwriting Movement Kinematics: From an Ecological to a Magnetic Resonance Environment.” Frontiers in Human Neuroscience 10: 488. 10.3389/fnhum.2016.00488. 27746727 PMC5040726

[hbm70172-bib-0005] Buneo, C. A. , and R. A. Andersen . 2006. “The Posterior Parietal Cortex: Sensorimotor Interface for the Planning and Online Control of Visually Guided Movements.” Neuropsychologia 44: 2594–2606. 16300804 10.1016/j.neuropsychologia.2005.10.011

[hbm70172-bib-0006] Chokron, S. 2003. “Right Parietal Lesions, Unilateral Spatial Neglect, and the Egocentric Frame of Reference.” NeuroImage 20: S75–S81. 10.1016/j.neuroimage.2003.09.002. 14597299

[hbm70172-bib-0007] Curzel, F. , S. Brigadoi , and S. Cutini . 2021. “fNIRS & e‐Drum: An Ecological Approach to Monitor Hemodynamic and Behavioural Effects of Rhythmic Auditory Cueing Training.” Brain and Cognition 151: 105753. 34020165 10.1016/j.bandc.2021.105753

[hbm70172-bib-0008] Cutini, S. , S. B. Moro , and S. Bisconti . 2012. “Review: Functional Near Infrared Optical Imaging in Cognitive Neuroscience: An Introductory Review.” Journal of Near Infrared Spectroscopy 20: 75–92.

[hbm70172-bib-0009] De Jong, B. M. , K. L. Leenders , and A. M. J. Paans . 2002. “Right Parieto‐Premotor Activation Related to Limb‐Independent Antiphase Movement.” Cerebral Cortex 12, no. 11: 1213–1217. 10.1093/cercor/12.11.1213. 12379609

[hbm70172-bib-0010] Delpy, D. T. , M. Cope , P. van der Zee , S. Arridge , S. Wray , and J. S. Wyatt . 1988. “Estimation of Optical Pathlength Through Tissue From Direct Time of Flight Measurement You May Also Like Accuracy Enhancement in Reflective Pulse Oximetry by Considering Wavelength‐Dependent Pathlengths.” Physics in Medicine & Biology 33, no. 12: 1433. 3237772 10.1088/0031-9155/33/12/008

[hbm70172-bib-0011] Ehrsson, H. H. , J. P. Kuhtz‐Buschbeck , and H. Forssberg . 2002. “Brain Regions Controlling Nonsynergistic Versus Synergistic Movement of the Digits: A Functional Magnetic Resonance Imaging Study.” Journal of Neuroscience 22, no. 12: 5074–5080. 12077202 10.1523/JNEUROSCI.22-12-05074.2002 PMC6757747

[hbm70172-bib-0012] Fink, G. R. , J. C. Marshall , P. W. Halligan , et al. 1999. “The Neural Consequences of Conflict Between Intention and the Senses.” Neuroreport 29: 917–923. 10.1093/brain/122.3.497 10094258

[hbm70172-bib-0013] Franz, E. A. , H. N. Zelaznik , and G. Mccabe . 1991. “Spatial Topological Constraints in a Bimanual Task.” Acta Psychologica 77, no. 2: 137–151. 1759589 10.1016/0001-6918(91)90028-x

[hbm70172-bib-0014] Garbarini, F. , F. D'Agata , A. Piedimonte , et al. 2014. “Drawing Lines While Imagining Circles: Neural Basis of the Bimanual Coupling Effect During Motor Execution and Motor Imagery.” NeuroImage 88: 100–112. 24188808 10.1016/j.neuroimage.2013.10.061

[hbm70172-bib-0015] Huppert, T. J. , S. G. Diamond , M. A. Franceschini , and D. A. Boas . 2009. “HomER: A Review of Time‐Series Analysis Methods for Near‐Infrared Spectroscopy of the Brain.” Applied Optics 48, no. 10: D280–D298. 19340120 10.1364/ao.48.00d280 PMC2761652

[hbm70172-bib-0016] Kennedy, D. M. , J. B. Boyle , C. Wang , and C. H. Shea . 2016. “Bimanual Force Control: Cooperation and Interference?” Psychological Research 80: 34–54. 25481636 10.1007/s00426-014-0637-6

[hbm70172-bib-0017] Lee, D. Y. , E. H. Seo , I. H. Choo , et al. 2008. “Neural Correlates of the Clock Drawing Test Performance in Alzheimer's Disease: A FDG‐PET Study.” Dementia and Geriatric Cognitive Disorders 26: 306–313. 18841015 10.1159/000161055

[hbm70172-bib-0018] Lloyd‐Fox, S. , A. Blasi , and C. E. Elwell . 2010. “Illuminating the Developing Brain: The Past, Present and Future of Functional Near Infrared Spectroscopy.” Neuroscience and Biobehavioral Reviews 34: 269–284. 19632270 10.1016/j.neubiorev.2009.07.008

[hbm70172-bib-0019] Lucci, G. , M. Berchicci , D. Spinelli , and F. Di Russo . 2014. “The Motor Preparation of Directionally Incompatible Movements.” NeuroImage 91: 33–42. 24440527 10.1016/j.neuroimage.2014.01.013

[hbm70172-bib-0020] Matsuoka, T. , J. Narumoto , K. Shibata , et al. 2011. “Neural Correlates of Performance on the Different Scoring Systems of the Clock Drawing Test.” Neuroscience Letters 487: 421–425. 21055445 10.1016/j.neulet.2010.10.069

[hbm70172-bib-0021] Molavi, B. , and G. A. Dumont . 2012. “Wavelet‐Based Motion Artifact Removal for Functional Near‐Infrared Spectroscopy.” Physiological Measurement 33: 259–270. 22273765 10.1088/0967-3334/33/2/259

[hbm70172-bib-0022] Ohashi, Y. , T. Kochiyama , K. Tsuneyoshi , Y. Ohigashi , T. Murai , and K. Ueda . 2018. “Functional Connectivity During Monitoring for Visuomotor Incongruence.” Neuroreport 29: 917–923. 29787449 10.1097/WNR.0000000000001053

[hbm70172-bib-0023] Oldfield, R. C. 1971. “The Assessment and Analysis of Handedness: The Edinburgh Inventory.” Neuropsychologia 9, no. 1: 97–113. 5146491 10.1016/0028-3932(71)90067-4

[hbm70172-bib-0024] Pashler, H. , J. Duncan , C. Fagot , et al. 1994. “Dual‐Task Interference in Simple Tasks: Data and Theory.” Psychological Bulletin 116, no. 2: 220–244. 10.1037/0033-2909.116.2.220. 7972591

[hbm70172-bib-0025] Raimo, S. , G. Santangelo , and L. Trojano . 2021. “The Neural Bases of Drawing. A Meta Analysis and a Systematic Literature Review of Neurofunctional Studies in Healthy Individuals.” Neuropsychology Review 31, no. 4: 689–702. 33728526 10.1007/s11065-021-09494-4 PMC8593049

[hbm70172-bib-0026] Rudisch, J. , S. Fröhlich , N. H. Pixa , D. F. Kutz , and C. Voelcker‐Rehage . 2023. “Bimanual Coupling Is Associated With Left Frontocentral Network Activity in a Task‐Specific Way.” European Journal of Neuroscience 58: 2315–2338. 37165733 10.1111/ejn.16042

[hbm70172-bib-0027] Rueda‐Delgado, L. M. , E. Solesio‐Jofre , D. Mantini , P. Dupont , A. Daffertshofer , and S. P. Swinnen . 2017. “Coordinative Task Difficulty and Behavioural Errors Are Associated With Increased Long‐Range Beta Band Synchronization.” NeuroImage 146: 883–893. 27771348 10.1016/j.neuroimage.2016.10.030

[hbm70172-bib-0028] Sadato, N. , Y. Yonekura , A. Waki , H. Yamada , and Y. Ishii . 1997. “Role of the Supplementary Motor Area and the Right Premotor Cortex in the Coordination of Bimanual Finger Movements.” Journal of Neuroscience 17, no. 24: 9667–9674. 9391021 10.1523/JNEUROSCI.17-24-09667.1997 PMC6573404

[hbm70172-bib-0029] Scholkmann, F. , and M. Wolf . 2013. “General Equation for the Differential Pathlength Factor of the Frontal Human Head Depending on Wavelength and Age.” Journal of Biomedical Optics 18: 105004. 24121731 10.1117/1.JBO.18.10.105004

[hbm70172-bib-0030] Serrien, D. J. , A. H. Pogosyan , and P. Brown . 2004. “Cortico‐Cortical Coupling Patterns During Dual Task Performance.” Experimental Brain Research 157: 79–84. 14968279 10.1007/s00221-003-1822-9

[hbm70172-bib-0031] Sherrill, K. R. , E. R. Chrastil , R. S. Ross , U. M. Erdem , M. E. Hasselmo , and C. E. Stern . 2015. “Functional Connections Between Optic Flow Areas and Navigationally Responsive Brain Regions During Goal‐Directed Navigation.” NeuroImage 118: 386–396. 26054874 10.1016/j.neuroimage.2015.06.009 PMC9441296

[hbm70172-bib-0032] Wenderoth, N. , F. Debaere , S. Sunaert , and S. P. Swinnen . 2005a. “Spatial Interference During Bimanual Coordination: Differential Brain Networks Associated With Control of Movement Amplitude and Direction.” Human Brain Mapping 26: 286–300. 15965999 10.1002/hbm.20151 PMC6871760

[hbm70172-bib-0033] Wenderoth, N. , F. Debaere , S. Sunaert , and S. P. Swinnen . 2005b. “The Role of Anterior Cingulate Cortex and Precuneus in the Coordination of Motor Behaviour.” European Journal of Neuroscience 22: 235–246. 16029213 10.1111/j.1460-9568.2005.04176.x

[hbm70172-bib-0034] Wenderoth, N. , F. Debaere , S. Sunaert , P. Van Hecke , and S. P. Swinnen . 2004. “Parieto‐Premotor Areas Mediate Directional Interference During Bimanual Movements.” Cerebral Cortex 14, no. 10: 1153–1163. 10.1093/cercor/bhh075. 15142955

[hbm70172-bib-0035] Zhao, H. , S. Brigadoi , D. Chitnis , et al. 2020. “A Wide Field‐Of‐View, Modular, High‐Density Diffuse Optical Tomography System for Minimally Constrained Three‐Dimensional Functional Neuroimaging.” Biomedical Optics Express 11: 4110. 32923032 10.1364/BOE.394914 PMC7449732

[hbm70172-bib-0036] Zhou, G. , Y. Chen , X. Wang , H. Wei , Q. Huang , and L. Li . 2022. “The Correlations Between Kinematic Profiles and Cerebral Hemodynamics Suggest Changes of Motor Coordination in Single and Bilateral Finger Movement.” Frontiers in Human Neuroscience 16: 957364. 10.3389/fnhum.2022.957364. 36061505 PMC9433536

[hbm70172-bib-0037] Zimeo Morais, G. A. , J. B. Balardin , and J. R. Sato . 2018. “FNIRS Optodes' Location Decider (fOLD): A Toolbox for Probe Arrangement Guided by Brain Regions‐of‐Interest.” Scientific Reports 8, no. 1: 3341. 10.1038/s41598-018-21716-z. 29463928 PMC5820343

