# Supplementary material for: Inferior Parietal Lobe Activity Reveals Bimanual Coupling and Interference
Source: Hum Brain Mapp. 2025 Feb 27;46(4):e70172. doi: 10.1002/hbm.70172 (PMC11867931; doi:10.1002/hbm.70172)
Supplement: Supplementary file 1 — Data S1. Supporting Information. [file HBM-46-e70172-s001.docx]

**Supplemental information**

*Global Analysis on deoxyhemoglobin concentration changes (HbR)*

Thirteen Brodmann Areas (BA) out of the eighteen were found to be active. Exploring differences in changes in HbR concentration, a significant main effect of the CONGR (F_(1,37)_ = 24.1, p<0.001) and BA factors (F_(12,444)_ = 15.73, p<0.001) and the interaction CONGR*BA (F_(12,444)_ = 6.85, p<0.001) were found. To enhance clarity in interpreting the HbR results, we will consider both increases and decreases in absolute terms. Thus, an elevation in HbR will signify heightened activity within the respective area, whereas a reduction will denote a return to baseline levels. The interaction effect revealed that several areas were significantly higher in the Incongruent condition compared to the Congruent one including premotor, sensorimotor and parietal areas (L-BA6, |-0.02|>|-0.015| µM, p<0.001; L-BA4, |-28.11|>|-22.06| µM, p<0.001; L-BA3, |-31.32|>|-22.50| µM, p<0.001; L-BA7, |-8.90|>|-5.78| µM, p=0.015; L-BA40, |-19.39|>|-14.92| µM, p<0.001; R-BA6, |-20.89|>|-16.35| µM, p<0.001; R-BA4, |-29.16|>|-21.85| µM, p<0.001; R-BA3, |-27.94|>|-17.35| µM, p<0.001; R-BA7, |-10.28|>|-4,72| µM, p<0.001 and R-BA40, |-19.39|>|-15.24| µM, p=0.001).

*Temporal Analysis on deoxyhemoglobin concentration changes (HbR)*

Fourteen BAs were found to be active in at least one bin. Thus, the non-active BAs (namely, L-BA10, L-BA9, L-BA8 and R-BA10) were excluded from the bin analysis.

Exploring differences among HbR concentration changes, a significant main effect of the BIN (F_(2,74)_=16.23, p<0.001), CONGR (F_(1,37)_=16.73, p<0.001) and BA factors (F_(13,481)_=14.94, p<0.001) was found. Moreover, the interactions BIN*CONGR (F_(2,74)_=9.38, p<0.001), BIN*BA(F_(26,962)_=13.21, p=0.039), CONGR*BA (F_(13,481)_=5.17, p<0.001) and BIN*CONGR*BA (F_(26,962)_=1.67, p=0.019) were found to be significant. To enhance clarity in interpreting the HbR results, we will consider both increases and decreases in absolute terms. Thus, an elevation in HbR will signify heightened activity within the respective area, whereas a reduction will denote a return to baseline levels. Post-hoc analysis revealed a significant reduction in HbR concentration changes as the HRF progressed in time, consistent with the HbO result (|bin 5-10s| > |bin 15-20s| and |bin 10-15s| > |bin 15-20s|) and a significant increasing in HbR concentration changes during the incongruent compared to congruent condition (|Incongr| > |Congr|). Moreover, in 5-10s bin a significant greater value in the Incongruent compared to the Congruent condition was found in L-BA6 (|-0.021|>|-0.015| µM, p<0.001), L-BA4 (|-0.032|>|-0.026| µM, p<0.001), L-BA3 (|-0.034|>|-0.027| µM, p<0.001), L-BA7 (|-0.01|>|-0.006| µM, p=0.006), L-BA39 (|-0.006|>|-0.002| µM, p<0.001), L-BA40 (|-0.021|>|-0.018| µM, p=0.007), R-BA6 (|-0.021|>|-0.015| µM, p<0.001), R-BA4 (|-0.031|>|-0.023| µM, p<0.001), R-BA3 (|-0.029|>|-0.019| µM, p<0.001), R-BA7 (|0.010|>|-0.004| µM, p<0.001) and R-BA40 (|-0.019|>|-0.013| µM, p<0.001).
